# Treatment of thoracogastric–mediastinal–pleural–airway fistula with esophageal stent, vascular plug, and tissue glue

**DOI:** 10.1055/a-2500-2869

**Published:** 2025-01-16

**Authors:** Yifan Li, Yahua Li, Binbin Ren, Zongming Li, Xinwei Han, Zhen Li, Kewei Ren

**Affiliations:** 1 191599 Department of Interventional Radiology, The First Affiliated Hospital of Zhengzhou University, Zhengzhou, China; 2 Medical 3D Printing Center of Henan Province, Zhengzhou, China

Thoracogastric airway fistula is a rare and serious complication post-esophageal cancer surgery with an incidence of about 0.2% to 1.9% 1 . Due to the complexity of these fistulas, endoscopic and conservative treatments often fail. Most patients are too frail for surgery, making intervention-based closure therapy a crucial treatment option 2 3 .

An 81-year-old man who had esophageal squamous cell carcinoma surgery over 6 years ago recently experienced choking while eating and gastrointestinal bleeding ( Fig. 1 ). A chest computed tomography (CT) scan showed a connection between the lower thoracogastric region and the mediastinum and airway in the chest ( Fig. 2 ).

**Fig. 1:**
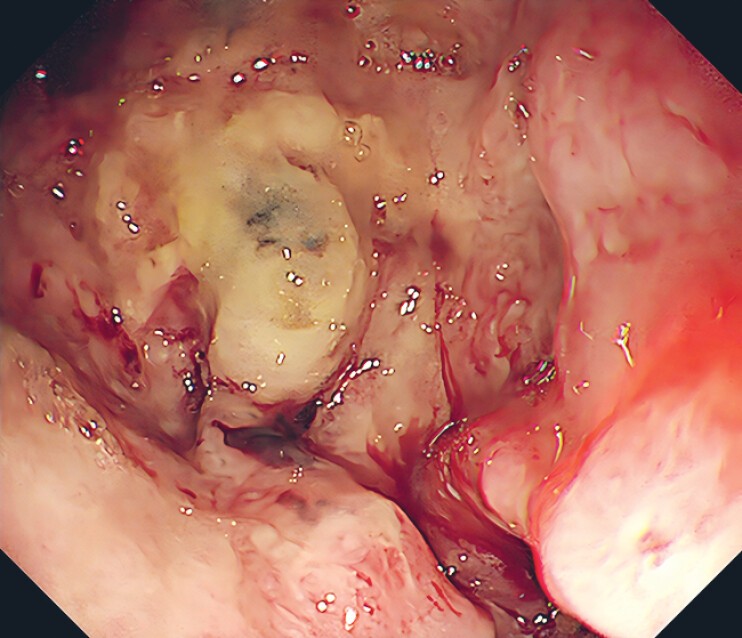
Fistula located in the lower thoracic stomach was observed via gastric endoscopy.

**Fig. 2:**
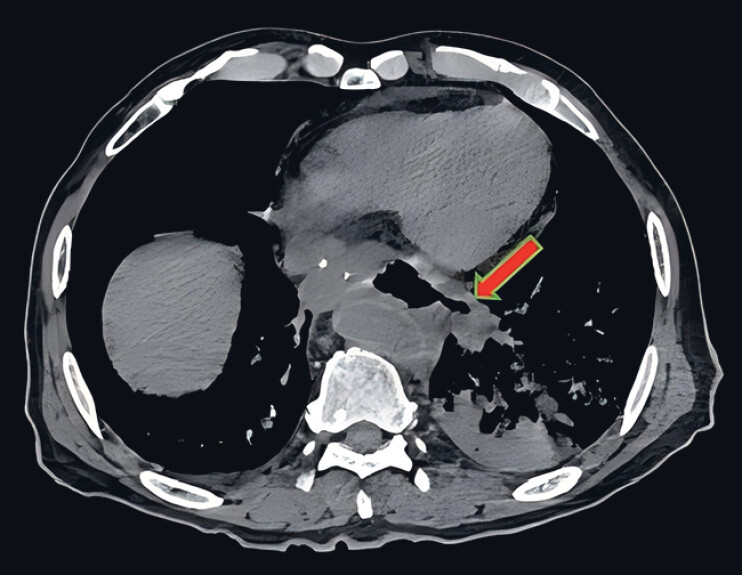
Preoperative chest computed tomography showed a thoracogastric tract connecting the left lower lobe bronchus through the mediastinum.

Angiography revealed thoracogastric contrast medium leaking into the mediastinum and the left lower lobe bronchus ( Video 1 ). A vertebral artery catheter was guided through the mediastinum into the bronchus of the fistula cavity. A stiff guidewire was then exchanged, followed by the insertion of an 8F sheath. A 6×6-mm vascular plug was deployed through the sheath to occlude the fistula tract. First, the initial segment was released on the tracheal side, followed by angiography to confirm its fit and blockage. Next, the second segment was released on the digestive tract side and inserted into a tissue glue and iodized oil emulsion (1:3). A pigtail catheter was placed in the mediastinal abscess cavity, and a water membrane with a hard guidewire was positioned in the jejunum. An esophageal stent (20×120 mm) and its delivery system were introduced and released after proper positioning ( Fig. 3 ). Finally, a jejunal nutrition tube was externally placed. The esophageal stent was removed 4 months after surgery due to displacement. Gastroscopic fistula healing was observed ( Fig. 4 ).

Final gastroscopic evaluation confirmed satisfactory fistula closure.Video 1

**Fig. 3:**
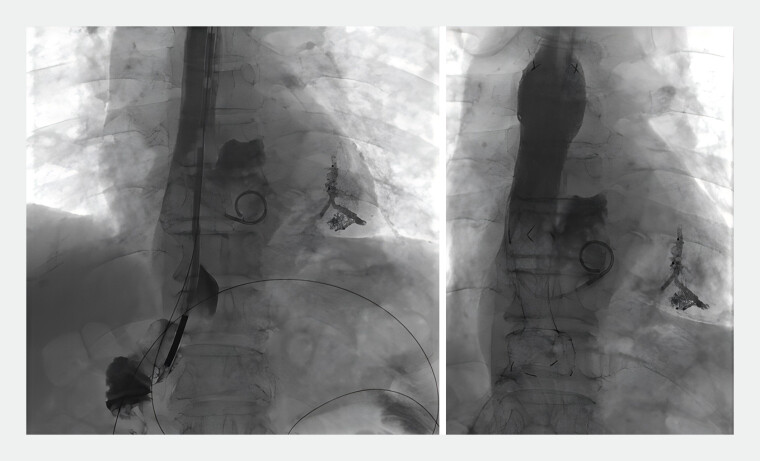
Post-occlusion fluoroscopy confirmed complete occlusion of the fistula tract, stabilization of the esophageal stent, vascular plug, and tissue glue, and visible abscesses and drains.

**Fig. 4:**
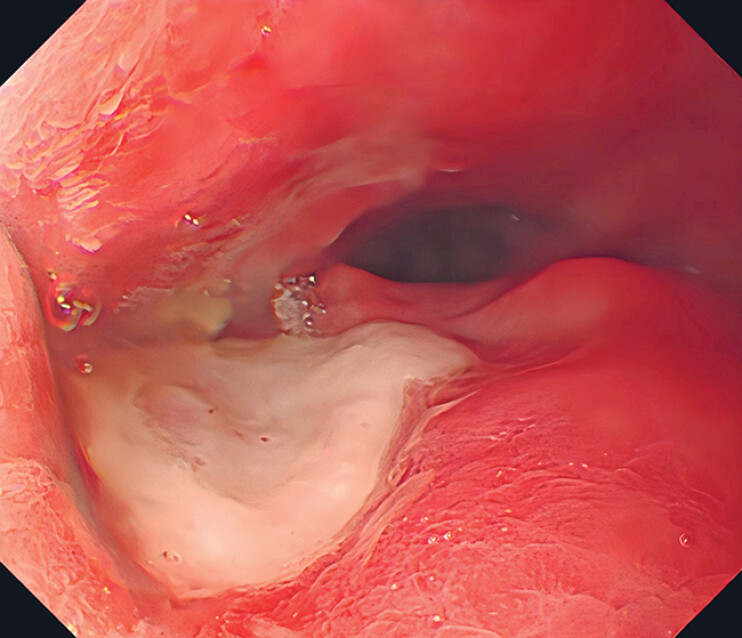
Gastroscopic evaluation demonstrated satisfactory closure of the fistula 5 months post-treatment.

Previous studies on tracheoesophageal fistula closure with Amplatzer devices primarily utilized occluders alone 2 4 . We propose using a vascular plug combined with tissue glue for complete fistula occlusion, offering new solutions for challenging cases that are unsuitable for stents or endoscopic methods.

Endoscopy_UCTN_Code_TTT_1AO_2AI

## Notes

Endoscopy E-Videos https://eref.thieme.de/e-videos
*E-Videos* is an open access online section of the journal *Endoscopy* , reporting on interesting cases and new techniques in gastroenterological endoscopy. All papers include a high-quality video and are published with a Creative Commons CC-BY license. Endoscopy E-Videos qualify for HINARI discounts and waivers and eligibility is automatically checked during the submission process. We grant 100% waivers to articles whose corresponding authors are based in Group A countries and 50% waivers to those who are based in Group B countries as classified by Research4Life (see: https://www.research4life.org/access/eligibility/ ). This section has its own submission website at https://mc.manuscriptcentral.com/e-videos .
